# Magnetic Mishaps—Small Bowel Obstruction Caused by Ingested Magnets Complicated by Appendicitis: A Case Report

**DOI:** 10.5811/cpcem.49053

**Published:** 2026-07-28

**Authors:** Seth Ball, Marissa Wierzbicki

**Affiliations:** Kirk Kerkorian School of Medicine at University of Nevada, Las Vegas, Department of Emergency Medicine, Las Vegas, Nevada

**Keywords:** pediatric ingestions, magnets, small bowel obstruction, appendicitis, case report

## Abstract

**Introduction:**

Magnet ingestions are a growing pediatric emergency due to the use of widely available stronger magnets in toys. A legislative ban briefly reduced injuries, but cases rose after its reversal in federal court. Magnet ingestions are resulting in increasing morbidity and mortality, specifically due to bowel obstruction and perforation.

**Case Report:**

We present a complex case involving a child with global developmental delay presenting to the emergency department for evaluation of abdominal pain. While the initial evaluation focused on working up appendicitis, which he was found to have, he was also found to have ingested multiple magnets causing small bowel obstruction and perforation.

**Conclusion:**

This case highlights the need for emergency physicians to maintain a high level of suspicion when evaluating pediatric patients with abdominal pain, especially those with developmental delay. It also encourages parental education and further advocacy to help stem the increase in pediatric morbidity and mortality resulting from the ingestion of magnets.

## INTRODUCTION

Foreign body ingestion is a common pediatric occurrence, with the vast majority passing spontaneously without intervention. Fewer than 1% require surgery, while 10–20% necessitate endoscopic retrieval.^1^–^2^ However, ingestion of magnets, especially multiple magnets, represents a unique subset of ingestions due to the high incidence of morbidity and mortality. The increasing frequency of such ingestions, coupled with the development of stronger magnets, has led to a steadily rising incidence of serious outcomes, including bowel perforation and obstruction.^1^ This necessitates a high index of suspicion on the part of the emergency physician along with prompt recognition and often surgical management.

Here we present the case of a 4-year-old boy with global developmental delay who returned to the emergency department (ED) one day after his initial presentation due to concern for appendicitis. Upon further evaluation, it was determined that in addition to appendicitis, the patient had multiple magnets attached across the bowel wall causing small bowel obstruction and multiple perforations.

## CASE REPORT

A 4-year-old boy with global developmental delay presented with two days of periumbilical pain and vomiting. The day prior to this evaluation, the patient was seen in the ED and, being found to have a soft abdomen without tenderness, was given ondansetron. He tolerated oral intake and was discharged with specific return precautions. He returned the following day with worsening symptoms that included right lower quadrant pain. He remained afebrile; his mother reported he had one bowel movement in the prior three days.

Physical examination showed a well-appearing male in no distress, mildly fatigued with periumbilical and right lower quadrant tenderness without peritoneal signs. Labs demonstrated mild leukocytosis to 14,000 per microliter (μL) (reference range: 4,000–11,000/μL); C-reactive protein, 32 milligrams per liter (mg/L) (< 3 mg/L), and an unremarkable comprehensive metabolic panel. Abdominal ultrasound showed a small amount of free fluid but did not visualize the appendix.

**Image 1:**
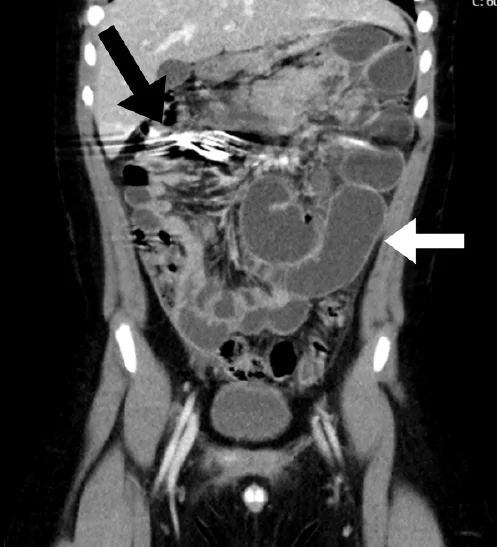
Computed tomography demonstrating streak artifact from ingested foreign bodies (black arrow) and dilated loops of bowel (white arrow) suggesting small bowel obstruction in a 4-year-old boy presenting with abdominal pain.

**Image 2:**
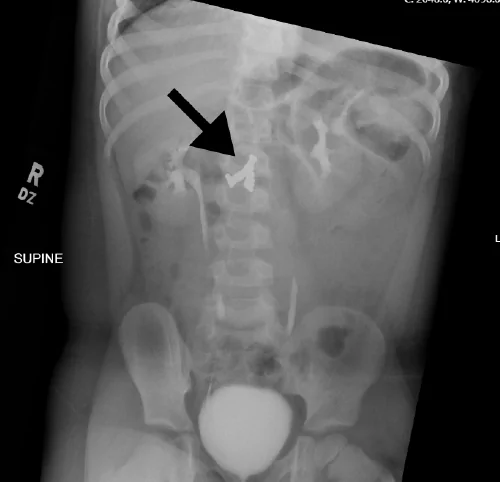
Radiograph demonstrating ingested stacked magnets (arrow).

Computed tomography (CT) of the abdomen and pelvis revealed acute appendicitis and small bowel obstruction caused by ingested stacked magnets with suspected bowel wall trapped between them (Image 1). Due to the finding of the magnets on CT, radiograph of the abdomen was also obtained, which more clearly demonstrated the stacked magnets (Image 2).

**Image 3:**
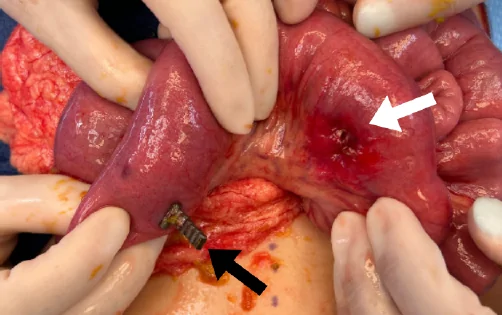
Intraoperative image demonstrating stacked magnets (black arrow) and perforation of the intestinal wall (white arrow) in a 4-year-old boy presenting with abdominal pain.

The patient underwent exploratory laparotomy where 15 magnets were found in the mid-jejunum causing five small bowel perforations (Image 3). A 3-cm bowel segment was resected.

The appendix was also removed due to mild inflammation. Pathology confirmed acute appendicitis with fecalith. The patient recovered well and was discharged after a short hospitalization.

*CPC-EM Capsule*

What do we already know about this clinical entity?

*Ingestion of high-powered magnets can cause bowel obstruction, ischemia, fistula formation, and intestinal perforation. It can be especially dangerous in children.*

What makes this presentation of disease reportable?

*This is a rare case of concurrent magnet-induced small bowel perforation and appendicitis in a non-verbal toddler, complicating diagnosis and management.*

What is the major learning point?

*In the case of a non-verbal child with apparent abdominal pain, keep the differential broad and consider additional injuries such as foreign body ingestion.*

How might this improve emergency medicine practice?

*This case highlights the need to broaden differentials in young children with abdominal pain and supports advocacy to limit access to such high-risk items.*

## DISCUSSION

Foreign body ingestion is a common presenting problem in the pediatric ED. While most foreign bodies will pass without complication, not all ingestions are benign. A variety of complications occur when the foreign body occludes a hollow viscus such as the appendix, the Meckel diverticulum, and the small bowel.^3^–^6^ Additionally, our case concerned a patient with developmental delay. Patients with autism spectrum disorder, intellectual disability, or other forms of developmental delay are at increased risk for serious complications.^6^–^9^ They often demonstrate more impulsivity and sensory-seeking behavior. This can lead to repeat presentations for foreign body ingestion as well as delayed presentations due to an inability or unwillingness to inform their caretakers.

Delayed presentation means the ingested foreign body is more likely to have progressed through the stomach and more likely to become lodged at anatomic narrowings such as the duodenojejunal flexure, mid-ileum, or ileocecal valve. Certain foreign bodies present a clear danger to this already vulnerable population, with magnets and button batteries being two culprits that carry high rates of morbidity and mortality. Not only are they two of the more dangerous, readily available consumer products, magnets, especially the newer, stronger, rare-earth metal magnets, can be part of children’s toys. These types of magnets are up to 30 times stronger than a typical magnet.^10^–^15^ A typical magnet can lead to strong attraction across the bowel wall leading to the bowel perforation in over 50% of cases of multiple magnet ingestion. Even a small, 5-mm magnet can exert up to half a kilogram of attraction force, which leads to severe gastrointestinal injuries such as ischemia, necrosis, and perforation, fistulas, obstruction, peritonitis, and even death.^10^

Between 2002–2011, there were an estimated 16,386 magnet ingestions in children < 18 years of age in the United States.^13^ In an attempt to reduce the number of injuries associated with ingestion of magnets, the U.S. Consumer Product Safety Commission initiated public awareness campaigns and a ban on high-powered magnets in 2014. Despite the initial success of these campaigns and the subsequent ban, the U.S. Court of Appeals for the Tenth Circuit overturned the ban in 2016, leading to a sharp rise in emergency visits related to magnet ingestion.^16^–^19^ Foreign body ingestion most commonly affects children six months–six years of age, but magnet ingestions specifically exhibit a biphasic age distribution. This is due to a second peak in older children and teens who often use magnets to mimic facial piercings and can accidentally ingest these imitation piercings.

## CONCLUSION

Magnet ingestions represent a special subset of pediatric ingestions that require the emergency physician to maintain a high index of suspicion, due to the rising number of incidents. and potential for increased morbidity and mortality. This is especially important in the patient who is unable to provide a concise history. Given the initial success of the 2014 public awareness campaign and ban on these magnets, further advocacy and parental education should be emphasized.
